# Combining PCR to detect junction fragments and deleted exons in the prenatal diagnosis of BMD can effectively identify maternal cell contamination

**DOI:** 10.3389/fmed.2025.1599498

**Published:** 2025-09-03

**Authors:** Lidan Cai, Wei Li, Min Zhong

**Affiliations:** ^1^Department of Psychiatry and Psychology, Nanfang Hospital, Southern Medical University, Guangzhou, Guangdong, China; ^2^Department of Neurology, Nanfang Hospital, Southern Medical University, Guangzhou, Guangdong, China; ^3^Department of Emergency, Nanfang Hospital, Southern Medical University, Guangzhou, Guangdong, China; ^4^Nanfang Hospital Baiyun Branch, Southern Medical University, Guangzhou, Guangdong, China

**Keywords:** Becker muscular dystrophy, DMD gene, junction fragments, maternal cell contamination, prenatal diagnosis

## Abstract

**Background:**

The junction fragment after the *DMD* gene deletion has been identified as a new specific DNA sequence formed by the reconnection of the ends. Our study aims to report a novel method for prenatal diagnosis of BMD by using PCR to detect junction fragments and deleted exons.

**Methods:**

We performed the prenatal diagnosis of a fetus with deletional BMD in this study. The proband of this family was the deletion of exons 3 to 5 of the *DMD* gene. The junction fragment primer designed after locating the breakpoint was used to PCR-amplify the junction fragments of the villus sample and the amniotic fluid genomic DNA. The exon 3 primer was used to amplify the deletion exons of the *DMD* gene from the villus sample and the amniotic fluid genomic DNA, respectively. At the same time, sex identification was carried out. Finally, the diagnosis results were analyzed.

**Results:**

The diagnosis of villus sampling was a contradictory result of obtaining the *DMD* gene deletion junction fragment and the absence of the exon deletion in the male fetus, suggesting that the villus sample was contaminated by maternal cells and the test was unsuccessful. The subsequent diagnosis of amniotic fluid was that the male fetus detected both the junction fragment and the corresponding exon deletion, and was diagnosed as a male fetus with BMD.

**Conclusions:**

Combining PCR to detect junction fragments and deleted exons in the prenatal diagnosis of BMD can effectively identify maternal cell contamination. The results were confirmed to be highly accurate and specific.

## 1 Introduction

Becker muscular dystrophy (BMD) is a common X-linked recessive genetic disease that primarily affects muscle tissue. BMD and Duchenne muscular dystrophy (DMD) are both considered allelic diseases that differ in severity and are caused by mutations in the *DMD* gene located in Xp21 (1). The large deletion mutation in the *DMD* gene has been determined to be responsible for the majority of DMD/BMD cases (~65%) (2). After deletion mutations are induced in the *DMD* gene, they are almost repaired by non-homologous end joining (3). In this process, the resulting junction fragment represents a new specific DNA sequence formed by the reconnection of the ends. Previously, under the condition that the full sequence of the *DMD* gene was basically clear, we have used a polymerase chain reaction (PCR)-based genome-walking method to accurately locate the missing breakpoint and we have directly amplified the junction fragment sequence by PCR (4).

The overall incidence of DMD/BMD is high, with ~1 of every 3,500 live-born boys affected (5). However, no effective treatment is currently available (6, 7). Approximately two-third of DMD/BMD cases are derived from familial inheritance (8, 9); thus, the prenatal diagnosis of pregnant women in DMD/BMD families is important to prevent the birth of a sick fetus and is currently the most effective way to reduce the incidence of DMD/BMD (10, 11). Commonly used detection technologies, such as multiplex PCR (12, 13), quantitative PCR (14, 15), fluorescence *in situ* hybridization (FISH) (16, 17), multiplex amplifiable probe hybridization (MAPH) (2, 18), and multiplex ligation-dependent probe amplification (MLPA) (19, 20), have been applied in the past decade to DMD/BMD pathogenic gene detection. However, prenatal diagnosis requires methods with greater detection ability. Prenatal diagnosis typically uses collected test specimens such as villus tissue, amniotic fluid, and umbilical cord blood; however, the small amount and possible maternal cell contamination presents difficulties in the detection and may interfere with the accuracy of the diagnostic results (21, 22). The aforementioned MLPA, FISH, quantitative PCR, etc. can be used to detect and analyse pathogenic genes in the prenatal diagnosis of DMD/BMD, but when the test specimen is contaminated with maternal cells, it cannot be additionally identified, which affects the accuracy of the test results. Therefore, laboratory testing of maternal cell contamination of each prenatal diagnostic sample has become a standard part of prenatal diagnosis (23). In this study, we diagnosed the fetus of a pregnant woman who is a carrier of BMD by detecting the missing exons, while simultaneously detecting the sequence of the junction fragment. We found that for male fetuses, maternal cell contamination can be effectively identified while achieving a prenatal diagnosis of DMD/BMD. However, for female fetuses, due to the fact that the mother is a female carrier, it is difficult to accurately identify whether the specimen being tested is contaminated by maternal cells using this method. Therefore, it is impossible to distinguish whether the fetus is a normal female or a carrier, which is the limitation of this method.

## 2 Materials and methods

### 2.1 Case information

**Figure 1:**
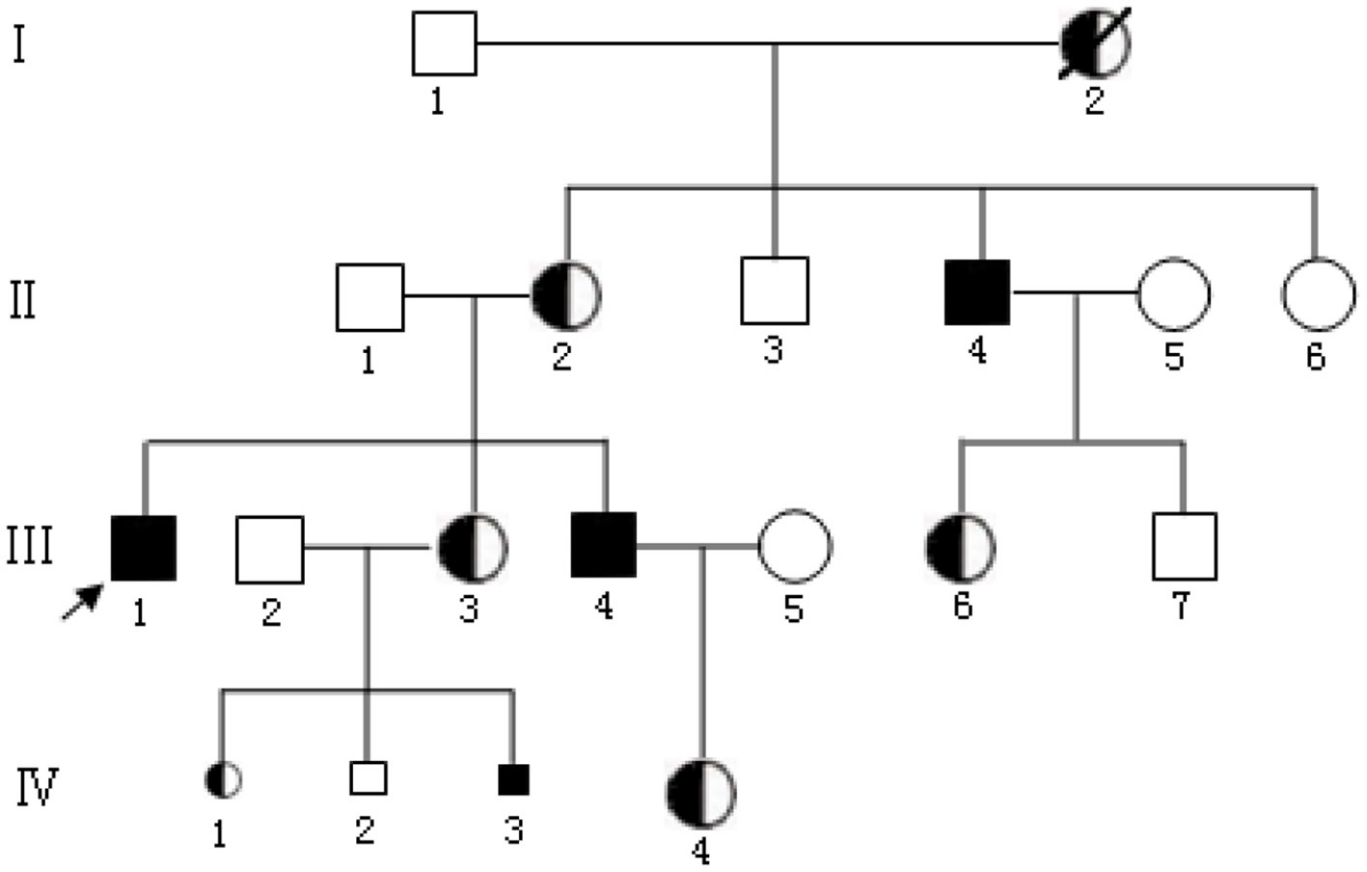
Pedigree of a BMD family.

Family members of a BMD family from Zhaoqing, Guangdong Province, China comprised the participants in this study. Proband III_1_ was a clinically diagnosed BMD patient and the deletion of exons 3 to 5 in the *DMD* gene were confirmed in this individual. After confirming that the 28 years old female III_3_ in the family was a carrier, she provided written informed consent to become a research participant in this study. Carrier III_3_ was provided genetic counseling and had three pregnancies, with the first two ending in abortion due to accidents. IV_3_ was the third fetus to be diagnosed at pregnancy. A villus sample was obtained (about 30 mg) at 11 weeks of pregnancy and an amniotic fluid sample (20 ml) was obtained at 20 weeks of pregnancy for prenatal genetic diagnosis (Figure 1).

### 2.2 Identification of proband genotype and carrier detection

In this family, genomic DNA was extracted from proband III_1_ and possible carriers III_3_ by proteinase K and phenol extraction. For PCR detection, 18 exon primers (12, 24), in conjunction with primers from the second and fifth exons [refer to the primer design provided by Bakker and Kneppers on the Leiden muscular dystrophy web page (http://www.dmd.nl)], were used and the proband was identified as the *DMD* gene deletion of exons 3 to 5.

After determining the extent of the exon deletion, the breakpoint was located at the corresponding intron by the PCR walking method of primer design involved the design of 5 pairs of primers in the second intron with an average sequence of about 30 kb. After detecting the region of the breakpoint in the first PCR reaction, we continued to design 1 pair of primers per 3 kb sequence in this region. In the fifth intron, we continued to directly design 1 pair of primers per 3 kb sequence. Each primer was designed to limit the length of its PCR product to between 301 and 400 bp. Using the above primers, the PCR reaction was carried out to locate the breakpoint. The 5′ end of the breakpoint was located at a site about 153 kb away from the first base in intron 2, while the 3′ end of the breakpoint was located near exon 6 in intron 5. A pair of primers (D1-F/R) was subsequently designed near the breakpoints of the two introns to directly amplify the junction fragment. The product fragment of about 2 kb was obtained and sequenced after purification. This junction fragment has been accepted and published in GenBank with serial number EF434728. The sequences of introns 2 and 5 of the *DMD* gene were obtained from http://www.dmd.nl/seqs.

After confirming the detailed sequence of the junction fragment of proband III_1_, the pair of primers (D4-F/R) for amplifying the junction fragment was redesigned to reduce the length of the PCR product to 495 bp, which improved the success rate of the junction fragment amplification compared with conventional PCR. The peripheral blood genomic DNA of III_3_ was then amplified using PCR with the D4-F/R primer pair and the 495-bp positive product fragment result was confirmed by sequencing. III_3_ of this family was subsequently diagnosed as a BMD female carrier.

### 2.3 Prenatal diagnosis

Genomic DNA was prepared immediately after samples for the prenatal diagnosis were collected. The villus samples were collected at the 11th week of pregnancy and were repeatedly washed with normal saline. The washed small pieces of tissue were then ground and crushed and the genomic DNA was extracted by the proteinase K and phenol method. The amniotic fluid sample (20 ml) was collected at the 20th week of pregnancy and was immediately centrifuged to collect the precipitate. The genomic DNA was extracted by the protease K and phenol method.

The primer was used to PCR-amplify the junction fragments of the villus sample and the amniotic fluid genomic DNA, with the junction fragment of Proband III_1_ used as a control. The primer (12) was used to amplify exon 3 of the *DMD* gene from the villus sample and the amniotic fluid genomic DNA, respectively. The ex6-F/R primer (12) was used to amplify the non-deleted exon 6 as a normal control to determine if there was an exon deletion corresponding to the proband. The sex identification primers, SRY-109F and SRY-245R (25), were used to identify the genomic DNA of the villus samples and amniotic fluid cells.

If a positive result was obtained from the above samples by amplifying the junction fragments and the corresponding deletion of the associated exons was found, the PCR products were further sequenced for verification.

### 2.4 Sequencing of the junction fragments

To verify the accuracy of the diagnostic results of this method, sequencing analysis was performed on the junction fragments of the proband III_1_, carrier III_3_, and the confirmed specimen from prenatal diagnosis. The aforementioned PCR products were sequenced after purification and electrophoresis observation. The sequencing work for this study was completed by Shanghai Invitrogen Biotechnology Co., Ltd., China, and the sequences of the PCR products were fully determined in both directions. The sequencing primers are shown in Appendix.

## 3 Results

### 3.1 Villus sampling diagnosis

**Figure 2:**
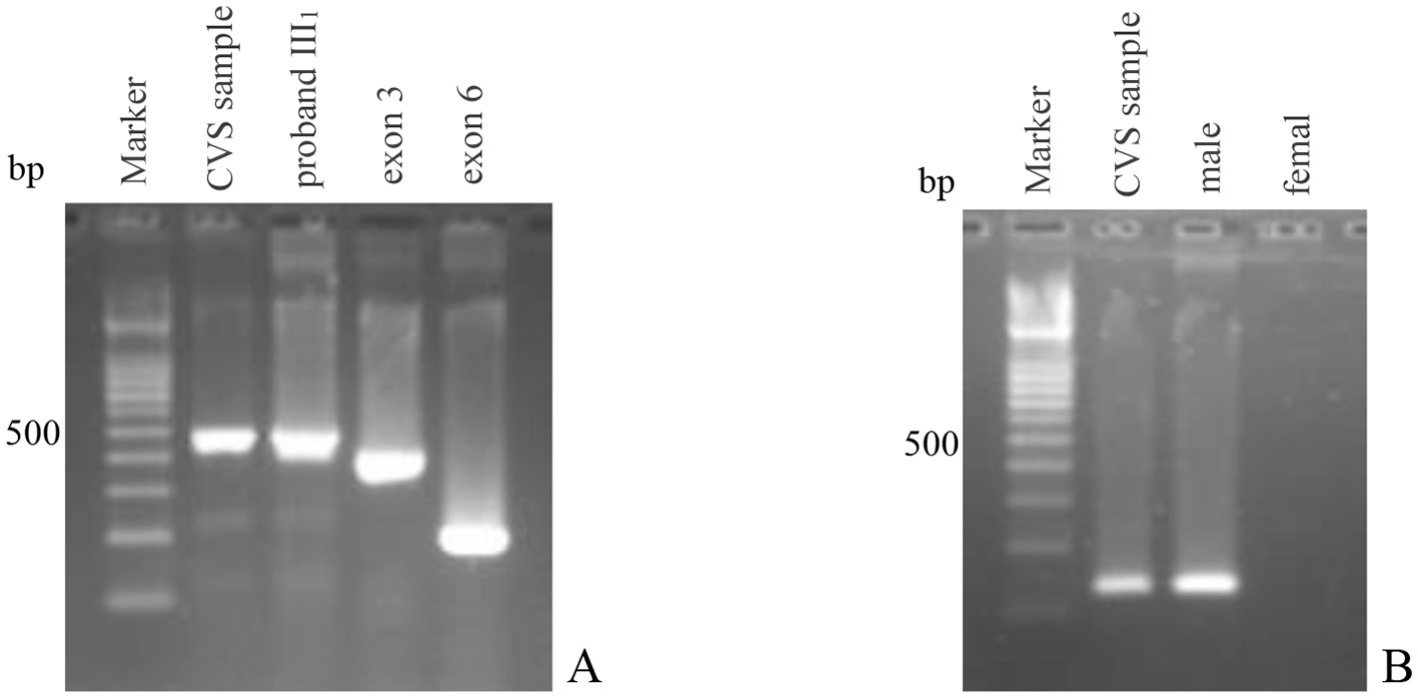
PCR diagnosis results of chorionic villi sampling of tertigravida III_3_. **(A)** PCR amplification of the junction fragment and the corresponding exons; Marker: 100 bp ladder marker; lane CVS sample: PCR amplification result of the junction fragment of the chorionic villi sample; lane proband III_1_: PCR amplification result of the junction fragment of proband III_1_; lane exon 3: PCR amplification result of exon 3 of the chorionic villi sample; lane exon 6: PCR amplification result of exon 6 of the chorionic villi sample; **(B)** PCR amplification of the *SRY* gene. Marker: 100 bp ladder marker; lane CVS sample: PCR amplification result of the *SRY* gene of the chorionic villi sample; lane male: normal male control; lane female: normal female control.

PCR results of the junction fragment from the genomic DNA of the villus tissue sample collected at the 11th week of pregnancy showed that a 495-bp product was amplified. This was the same length as the junction fragment product of the proband III_1_ of this family. However, testing of exon 3 showed a positive amplification without deletion. In addition, the gender was identified as male (Figure 2). The contradictory result of obtaining the *DMD* gene deletion junction fragment and the absence of the exon deletion in the male fetus indicated that the villus sample was contaminated by maternal cells and the test was unsuccessful.

### 3.2 Amniotic fluid diagnosis

**Figure 3:**
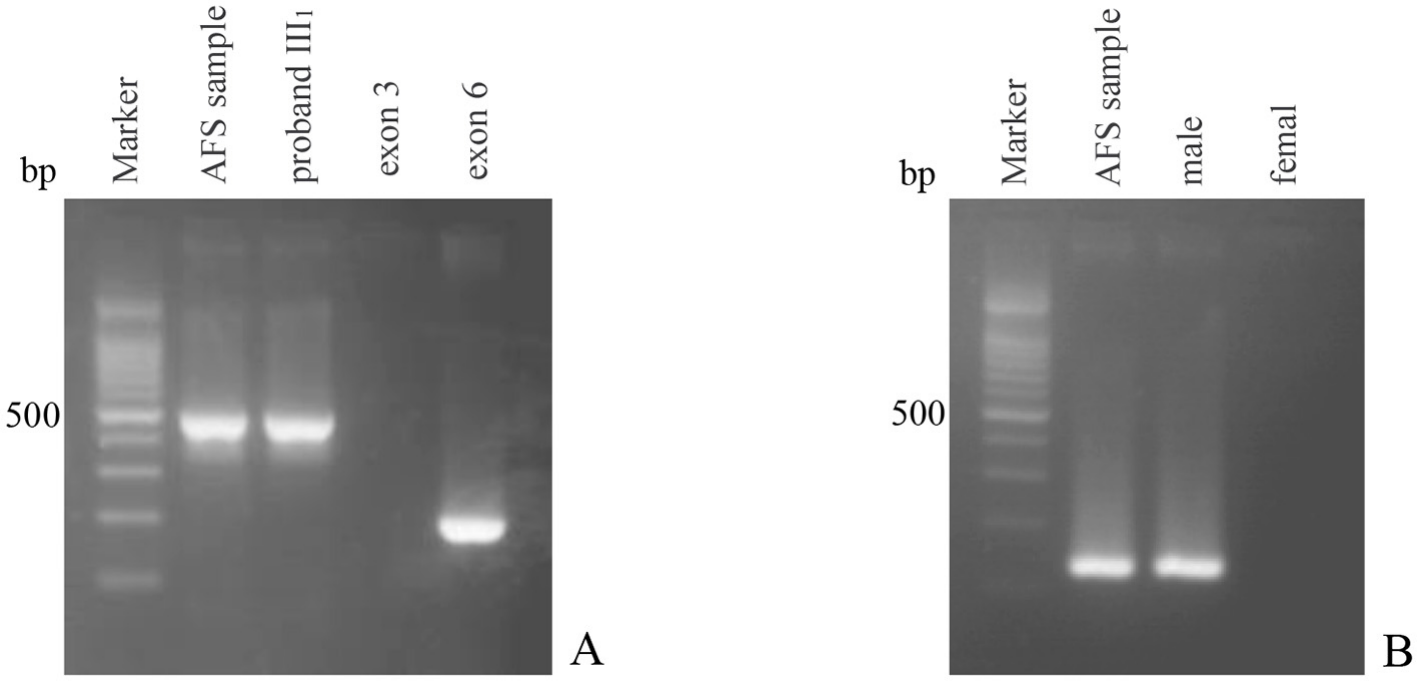
PCR diagnosis results of the amniotic fluid of tertigravida III_3_. **(A)** PCR amplification results of the junction fragment and the corresponding exons; Marker: 100 bp ladder marker; lane AFS sample: PCR amplification result of the junction fragment of the amniotic fluid sample; lane proband III_1_: PCR amplification result of the junction fragment of proband III_1_; lane exon 3: PCR amplification result of exon 3 of the amniotic fluid sample; lane exon 6: PCR amplification result of exon 6 of the amniotic fluid sample; **(B)** PCR amplifications of the *SRY* gene. Marker: 100 bp ladder marker; lane AFS sample: PCR amplification result of the *SRY* gene of the amniotic fluid sample; lane male: normal male control; lane female: normal female control.

PCR results of the junction fragment from the genomic DNA of the amniotic fluid cells taken at the 20th week of pregnancy showed that the 495-bp product was amplified, which was consistent with the length of the fragment product of the proband III_1_ of this family. The amplification of exon 3 was negative, demonstrating that the corresponding *DMD* gene region was deleted. The gender identification results were the same, indicating the fetus was male (Figure 3). Therefore, the fetal IV_3_ was diagnosed as a male fetus with BMD. Given this information, the pregnant woman III_3_ chose to terminate the pregnancy. The fetal tissue was subsequently analyzed and the diagnostic results were confirmed.

### 3.3 Sequencing results

**Figure 4:**
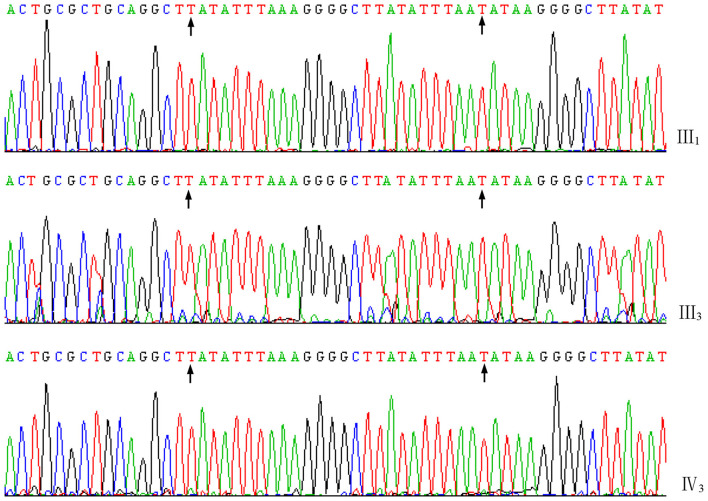
Forward sequences of the junction fragments of III_1_, III_3_, and IV_3_ of the family. The characters between the arrows indicate the 26 bp nucleotides inserted in the joint.

A total of 2,113 bp of valid sequences were obtained from the sequencing of the PCR products of proband III_1_, while 495 bp of valid sequences were obtained from the sequencing of the PCR products of carrier III_3_ and the amniotic fluid sample for prenatal diagnosis. These sequences were all confirmed to be the deletion junction fragments of the *DMD* gene through alignment by BLAST (https://blast.ncbi.nlm.nih.gov/Blast.cgi). Meanwhile, the sequencing results of the junction fragment in the amniotic fluid sample showed no difference from those of the junction fragments in proband III_1_ and carrier III_3_ in this family (Figure 4), indicating that they have the same genotype. The pathogenic gene is a new junction sequence formed by the insertion of a 26-bp sequence (TATATTTAAAGGGGCTTATATTTAAT) after the large fragment deletion of exons 3-5 of the *DMD* gene, which further verified the PCR diagnosis result that fetus IV_3_ is a male fetus with BMD.

## 4 Discussion

Female carriers of DMD/BMD have a 50% risk of passing the disease-causing mutant gene onto their offspring. Among the potential offspring, boys who acquire the disease gene will become sick, while the girls will become healthy carriers. Therefore, the overall risk of giving birth to a sick infant is 25% (26). Therefore, the prenatal diagnosis of DMD/BMD is necessary to provide the family with accurate and timely information regarding the health of the unborn fetus. Accordingly, the prenatal diagnosis of DMD/BMD necessitates 100% accuracy, with a highly specific diagnosis method. In general, the prenatal diagnosis of DMD/BMD is based on invasive methods (27, 28) and uses test specimens such as villus tissue, amniotic fluid, or umbilical cord blood to directly analyze the fetus (29, 30). The diagnostic results are reliable and considered the gold standard for prenatal diagnosis. However, due to the risk of maternal cell contamination in these specimens, the diagnostic results may still be incorrect (21, 31, 32). Therefore, the identification and exclusion of maternal cells during the prenatal diagnosis process remains an obstacle that most laboratories have been unable to avoid. However, because it can lead to a false negative diagnosis, extraordinary measures must be taken to address this issue (21, 33). The identification of maternal cell contamination is generally performed by short tandem repeat polymorphism linkage analysis (34, 35). Based on the fact that the junction fragment is a specific DNA sequence belonging to a patient or carrier with deletion DMD/BMD, we believe that a diagnosis method using PCR to detect both the junction fragment and the corresponding deleted exons can effectively identify the maternal cell pollution while simultaneously detecting the pathogenic gene. In this regard, the accuracy and reliability of the prenatal diagnosis results can be improved.

Since DMD/BMD is a sex-linked inheritable disease, the method described in this study first performs sex identification of the embryo or fetuse. For female fetuses, the protocol is to record the experimental results and continue the pregnancy but inform the pregnant women that the fetus may be a carrier, something that can be verified after delivery. For male fetuses, if the junction fragment is amplified positively, suggesting that the same genotype as the proband, then theoretically the corresponding exons should be deleted (negative amplification) and the fetus would be considered to have DMB/BMD. If the junction fragment is positive and the corresponding exons are subsequently found to be deleted, it can be confirmed that the fetus has DMD/BMD. This information should be provided to the pregnant woman in a timely manner so that a decision can be made whether to terminate the pregnancy. If the woman chooses to terminate the pregnancy, the aborted fetal tissues should be collected and the DNA prepared for diagnostic verification.

Before the decision to terminate the pregnancy is made, if testing indicates that the junction fragment results are positive but the corresponding exons are detected without deletion (positive amplification), the test specimen can be considered to be contaminated with maternal cells and testing should be repeated. If the junction fragment results are negative and the corresponding exons are detected without deletion, the fetus can be considered to not have the junction fragment and there is no exon deletion corresponding to the proband. In the latter case, this represents a normal male fetus and the pregnancy can be continued without worry. In both cases, the results can be verified postpartum.

In this study, during the prenatal diagnosis of carrier III_3_, we performed villus sampling and amniotic fluid detection. The sex of the fetus was identified as male. A junction fragment consistent with Proband III_1_ was detected in the villus sample, but there was no detection of the associated deletion in the corresponding exons. This suggested that the specimen was contaminated with maternal cells and testing was repeated. In this process, the amniotic fluid sample was tested again and the junction fragment was still positive. However, the deletion of the corresponding exons was detected this time. These results ruled out the possibility of maternal cell contamination and confirmed the diagnosis of fetal IV3 with BMD. The probability of villus samples being contaminated with maternal cells during prenatal diagnostic sampling may be slightly higher than that of amniotic fluid samples (36). However, a disadvantage of amniotic fluid and umbilical cord blood analysis is that it can only be performed in the second trimester of pregnancy, making these types of samples not suitable for early diagnosis. Therefore, to achieve the best prenatal diagnosis results, laboratories need to complete the diagnosis as early as possible. At the same time, the diagnosis method should have procedures in place to effectively identify whether the specimen to be tested is contaminated with maternal cells (21, 37, 38).

While the method described in this study solves the problem of identifying whether the specimen to be tested is contaminated with maternal cells for male fetuses, it remains difficult to accurately identify whether the specimen to be tested is contaminated with maternal cells for female fetuses because the mother is a female carrier. For this reason, it is advised to wait for postpartum examination and verification when the fetus has been identified as female. In addition, the use of the junction fragment for DMD/BMD prenatal diagnosis requires a detailed analysis of the genotypes of the probands in the family, which has the disadvantage of requiring a number of complicated steps. Indeed, because the *DMD* gene sequence is extremely large and the mutation types are complex, each *DMD* gene detection method has their own advantages and disadvantages. For the actual application of these methods in the clinical setting, each laboratory is required to adapt their method to the specific circumstances of the case. For example, MAPH and MLPA technologies (18, 39), which are considered to be the most promising methods for detecting *DMD* gene deletions and repeated mutations, are not perfect techniques. In fact, studies have repeatedly identified these methods as time-consuming and cumbersome (40, 41), with a relatively high cost (42) and inability to apply to single cell analysis. In addition, many difficulties remain in terms of preimplantation genetic diagnosis (PGD) (26). In contrast, the method developed in this study only requires a small amount of test samples, making it feasible for application in PGD. Moreover, for male fetuses, this method has a distinctive advantage: it can effectively identify maternal cell contamination while performing prenatal diagnosis.

## 5 Conclusions

During the process of detecting the deleted exons and the junction fragments in two samples of villus and amniotic fluid via PCR, we found that maternal cell contamination can be effectively identified while conducting prenatal diagnosis for DMD/BMD in male fetuses. Our results have been confirmed to be highly accurate and specific. Overall, our findings indicate that this method for identifying maternal cell contamination during the diagnosis of male fetuses has a distinct advantage that can be implemented in the clinical setting. However, for female fetuses, it is still impossible to accurately distinguish between normal females and female carriers.

## Data Availability

The datasets presented in this study can be found in online repositories. The names of the repository/repositories and accession number(s) can be found in the article/supplementary material.

## Appendix

PCR primer sequences in introns 2 and 5:

2-B1F 5′- GTTTGCTGAATCTGCCTCTGT -3′

2-B1R 5′- GGCCTTACGTGACCTGACAT -3′

2-C1F 5′- CAGGCATGAAGGTTGCCTAT -3′

2-C1R 5′- TCTCACCCATTTTGCCACTC -3′

2-D1F 5′- CGGAAGGGAGCTTAGTAGCA -3′

2-D1R 5′- ACTGAATGGATTTCGGGATG -3′

2-E1F 5′- AGCAGCCCTCATTGCATATT -3′

2-E1R 5′- GAGCCAGTGGCGTTAAACAG -3′

2-F1F 5′- AGGCAGGGTAGGACAATCAC -3′

2-F1R 5′- AAATAATGGCTCGGTCCACA -3′

2-F2F 5′- GCCAACCCAGATTTAAGAAGC -3′

2-F2R 5′- CCATCCGAGTGTACCGACTT -3′

2-F3F 5′- TCGAATGCACATCTGTTTGA -3′

2-F3R 5′- GGAACACCGTCGTCTTCTTT -3′

2-F4F 5′- TGTGTGTGAGATCTTGCTTCG -3′

2-F4R 5′- GGCAAATCGTATGCACTCAA -3′

2-F5F 5′- CAGCATTGATGTTGCCTGTC -3′

2-F5R 5′- GCAATACCCTACCAAGCACAA -3′

2-F6F 5′- TTGATGCTTGCCATCTAGGA -3′

2-F6R 5′- ATGGCATTTCTGGACTCACC -3′

2-F7F 5′- GACCCATCACCTGACGTTTT -3′

2-F7R 5′- TTCACCAAATCGTTTTCTGC -3′

5-1F 5′- CCATGGTCCCTTCACTATCAA -3′

5-1R 5′- TTGAGAGGCTACCAAGAACCA -3′

5-2F 5′- TGCCCCTTCATCAGTCAATAG -3′

5-2R 5′- CCCAAAGGTGAGCCTGTAAA -3′

PCR primer sequences for the junction fragment:

D1-F 5′- AAATCAAGAGAAGGTTAATGTGGAC -3′

D1-R 5′- CTTACCTATGACTATGGATGAGAGCA -3′

D1-F2 5′- TGGGCAAGGAATACAACCTTA -3′

D1-F3 5′- ACACCTCTTAGGGATAAATA -3′

D1-R2 5′- AGGAGGAATACTATAGATAA -3′

D1-R3 5′- GCAACCTCCGTCTCCCAGTG -3′

D4-F 5′- ATTTGAAAGTTGGCCGGGTA -3′

D4-R 5′- TCATAGGCTTCGTGCATGTG -3′
